# Effects of extreme weather events on child mood and behavior

**DOI:** 10.1111/dmcn.14856

**Published:** 2021-03-15

**Authors:** Jennifer L Barkin, Massimiliano Buoli, Carolann Lee Curry, Silke A von Esenwein, Saswati Upadhyay, Maggie Bridges Kearney, Katharine Mach

**Affiliations:** ^1^ Mercer University School of Medicine Macon GA USA; ^2^ Department of Neurosciences and Mental Health Fondazione IRCCS Ca’ Granda Ospedale Maggiore Policlinico Milan Italy; ^3^ Department of Pathophysiology and Transplantation University of Milan Milan Italy; ^4^ Rollins School of Public Health Emory University Atlanta GA USA; ^5^ University of Miami Rosenstiel School of Marine and Atmospheric Science Miami FL USA; ^6^ Leonard and Jayne Abess Center for Ecosystem Science and Policy University of Miami Coral Gables FL USA

## Abstract

Extreme weather events (EWEs) are increasing in frequency and severity as the planet continues to become warmer. Resulting disasters have the potential to wreak havoc on the economy, infrastructure, family unit, and human health. Global estimates project that children will be disproportionately impacted by the changing climate – shouldering 88% of the related burdens. Exposure to EWEs in childhood is traumatic, with ramifications for mental health specifically. Symptoms of posttraumatic stress, depression, and anxiety have all been associated with childhood EWE exposure and have the potential to persist under certain circumstances. Conversely, many childhood survivors of EWE also demonstrate resilience and experience only transient symptoms. While the majority of studies are focused on the effects resulting from one specific type of disaster (hurricanes), we have synthesized the literature across the various types of EWEs. We describe psychological symptoms and behavior, the potential for long‐term effects, and potential protective factors and risk factors.

**What this paper adds**

- Climate change‐related phenomena such as extreme weather events (EWEs) have the potential to impact mood and behavior in children.
- Posttraumatic stress (PTS) is the most common mental health consequence in child survivors of EWEs.
- PTS is often comorbid with depression and/or anxiety in this group.

## What this paper adds

- Climate change‐related phenomena such as extreme weather events (EWEs) have the potential to impact mood and behavior in children.
- Posttraumatic stress (PTS) is the most common mental health consequence in child survivors of EWEs.
- PTS is often comorbid with depression and/or anxiety in this group.

**Abbreviations**
EWE: Extreme weather event
PTG: Posttraumatic growth
PTS: Posttraumatic stress
PTSD: Posttraumatic stress disorder

Extreme weather events (EWEs), such as extreme heat, drought, flooding, wildfires, and storms, will continue to increase in frequency and severity as the planet becomes warmer.^1^ This is significant, as EWEs can have a devastating impact on individuals’ financial security, the economy as a whole (including the agricultural and housing sectors), and human health.^2^ Injury, illness, death, and population displacement are all potential consequences of EWEs that can cause mental health problems in affected individuals.^3^ Global estimates project that children will bear the burden of climate change,^4^, ^5^ with those living in poverty being the most affected.^6^ Specific to mental health, exposure to EWEs is often traumatic and has been associated with adverse psychosocial outcomes and developmental delays in adolescents.^7^

Both prevalent and unique findings related to the mental health of childhood EWE survivors are discussed within this narrative review. While the majority of articles are focused on the mental health effects resulting from one specific type of disaster (i.e. Hurricane Katrina), we have synthesized the literature across the various types of EWEs, highlighting commonalities and novel, but compelling, findings throughout. Psychological symptoms and behavior, the potential for long‐term effects, and potential protective or risk factors are described as they apply to the recent literature.

## PSYCHOLOGICAL SYMPTOMS AND BEHAVIOR

Posttraumatic stress (PTS) symptoms are a central focus of the EWE‐related mental health literature,^8^ and with justification: up to 71% of children experience PTS symptoms as the result of exposure to disasters.^9^, ^10^ It is important to note that whilst developing this article, the authors observed a variety of naming conventions for PTS throughout the body of literature; PTS, posttraumatic stress disorder (PTSD), and posttraumatic symptoms were all used to describe post‐disaster trauma. This variance in terminology was maintained in the present article in order to remain true to the authors’ descriptions.

However, despite a focus on PTS, there are a range of other psychiatric symptoms and behaviors that can manifest in children in the aftermath of an EWE.^11^ In fact, elevated rates of depression (which is highly comorbid with PTSD) have been observed in numerous studies and across EWE type.^12^, ^13^, ^14^, ^15^ A recent example is Hurricane Maria, the category 5 hurricane that devastated Puerto Rico in 2017. Results from a large survey of Puerto Rican adolescents (*n*=226 808) revealed high levels of depression and PTSD 5 to 9 months after the hurricane.^12^ Similar to depression, anxiety is often comorbid with PTS in the context of EWE exposure.^14^, ^16^ Anxiety related to concerns about one’s existence, ‘existential anxiety’, was described as highly prevalent and related to both elevated PTS and depression in a study of 325 adolescents conducted after Hurricane Katrina.^14^

In addition to mood symptoms, externalizing behaviors have also been associated with childhood exposure to EWEs.^17^, ^18^ A study focused on the relationship between ambient temperature and externalizing behaviors such as aggression and delinquency underscores the potential ramifications of heat exposure.^17^ Specifically, a significant increase in aggressive behaviors in urban‐dwelling Californian adolescents was observed with rising average temperatures (per 1°C increment); this result was equivalent to 1 year 6 months to 3 years of delay in age‐related behavioral maturation. It provides a preview of the behavioral health impact of a climate warming with longer and more frequent heat spells.^19^ The link between EWE exposure and aggressive behaviors is not exclusive to temperature extremes. It has been documented in the context of other natural disasters, including tornadoes.^18^ Though associations between general trauma and adolescent substance abuse have been more firmly established,^20^ several reports have implicated EWE‐specific trauma as a risk factor for substance abuse.^21^, ^22^, ^23^

Childhood disaster exposure is also associated with attachment security.^20^, ^24^ A study of children affected by the 1983 Ash Wednesday bushfires in Australia is illustrative. Findings indicated that even a brief separation of children from their parents during the event increased the risk of their developing an avoidant attachment style as an adult.^24^ This finding is consistent with the basic tenets of attachment theory, which asserts that the absence of attachment figures during a childhood crisis may contribute to adult attachment insecurity.^25^ It also has a broader application, as wildfires are among several types of EWEs with the potential to cause upheaval and displacement within the family unit and social support system.^7^ Likewise, presence of parents at the time of a disaster was identified as protective with respect to adolescent substance abuse in a study of the Spring of 2011 tornadoes in the United States.^21^

The impact on childhood learning is an additional and important consideration in the aftermath of an EWE. Diminished academic performance has been observed in EWE‐affected children and appears to be correlated with the degree/severity of exposure and resultant emotional distress.^26^, ^27^ For example, findings from a large study of school‐aged children (*n*=24 642) impacted by the 2009 bushfires in Victoria, Australia, demonstrated that those attending school in heavily affected areas exhibited diminished performance in reading and numeracy.^26^ Similarly, children who reported less distress/emotional symptoms in the wake of Hurricane Katrina reported fewer problems at school.^27^

Other potentially correlated behaviors/symptoms that figure less prominently in the recent literature include difficulty sleeping,^28^ diminished earning potential as an adult,^29^ lower function at home, post‐disaster suicidal ideation,^30^, ^31^ and suicide attempts.^31^

## POTENTIAL FOR LONG‐TERM EFFECTS

Multiple studies have demonstrated the potential for long‐term behavioral and mental health effects in children exposed to EWEs, and these effects have been observed across disaster types.^29^, ^32^, ^33^, ^34^, ^35^ The 2011 floods in Namibia are illustrative, as the majority of children in a study population of 480 reported symptoms of trauma 2 years later. Notably, the affected region, its residents, and schools had not fully recovered from previous floods in 2008 and 2009, which is likely to have compounded the mental health effect.^33^

A similar result was observed in rural Australian adolescents subject to prolonged drought.^32^ Specifically, adolescents who were evaluated several years after the drought began reported higher levels of emotional distress compared to individuals who were evaluated more proximal to the onset of the event.^32^, ^36^ The results also indicated new, self‐reported themes of grief and loss as the drought persisted, highlighting the impact of cumulative stress on both the individual and the community.^32^

Further, two separate studies of childhood tsunami survivors indicated that psychological symptoms have the potential to persist from months to years after the disaster. However, in both studies, the severity and type of exposure were associated with persistence of mood symptoms.^15^, ^31^ Longer‐term impacts have also been observed in studies of childhood survivors of tornadoes, hurricanes, and in utero heat exposure.^29^, ^34^, ^35^ It is important to note that despite the substantial body of evidence intimating the potential for negative long‐term effects of childhood EWE exposure, there are both protective and risk factors, such as resiliency, coping skills, and support networks that influence symptom and recovery trajectories.^37^ In short, the recovery process is not uniform; while some survivors exhibit persistent, negative mental health outcomes, many demonstrate resilience and experience only transient symptoms.^38^

## PROTECTIVE FACTORS

While PTS and general distress are often the primary mental health outcomes of interest in the disaster literature,^39^ recent reports have also focused on (potentially) protective factors and positive outcomes (as opposed to an exclusive focus on negative mood symptoms). These include coping efficacy and acculturation,^8^, ^40^ family resilience and social support,^7^ family functioning and posttraumatic growth (PTG).^41^ Of note, positive reappraisal, the coping technique whereby stressful events are recast as ‘benign, valuable, or beneficial’,^42^ was associated with increased family functioning and PTG in a study of parents and adolescents who had been evacuated due to the California coastal fires of 2008 and 2009.^41^ PTG is an indicator of the ability to adjust, progress, and reframe events in the wake of traumatic experiences. Similarly, dispositional mindfulness, the capacity to be mindful and attentive in the moment, was protective against PTSD and academic burnout in adolescent tornado survivors.^34^ This result is consistent with findings from a systematic literature review in which the authors cite a positive relationship between the practice of dispositional mindfulness and overall psychological health.^43^ Interestingly, the combination of high acculturation and high coping efficacy among black adolescents was found to be beneficial in at least one study related to the Florida wildfires of 1998.^8^

The evidence implicating social support as protective in the context of childhood EWE exposure is considerable and corroborated by the broader mental health literature.^44^, ^45^ Specifically, social support has the potential to mitigate the duration and severity of mood symptoms resulting from EWE exposure.^27^, ^46^, ^47^ Findings indicate that social support from parents, classmates, and friends correlates with a lower probability of developing and/or sustaining negative mental health effects post‐disaster.^16^, ^23^, ^46^ The importance of caregiver emotional support, in particular, has been cited in several recent studies,^24^, ^47^, ^48^ and also in relation to a decreased likelihood of binge drinking in adolescent tornado survivors.^47^ Further, the quality of the parent–child relationship was also inversely associated with both PTSD and degree of academic burnout in survivors of the tornado of 2016 in Yancheng, China. As a result, the authors suggest that clinical efforts to treat traumatized children should focus on optimizing the quality and function of the parent–child attachment.^49^ This assertion is supported in a study by Felix et al. who describe the protective influence of parent–child involvement in mitigating ‘ataques de nervios’ (attacks of the nerves) in Puerto Rican child survivors of Hurricane George.^50^

Additionally, the adoption of household air‐conditioning and high levels of neighborhood greenspace have also been identified as protective factors against the negative mental health effects associated with high heat exposure.^17^, ^29^

## RISK FACTORS

In addition to psychosocial and environmental factors, some demographic variables have been implicated when considering key influencers of post‐disaster mental health in children, although the results are somewhat mixed. For example, older age and being female have been associated with PTS, depressive symptoms, and negative externalizing behaviors in separate studies during which EWE survivors were evaluated; females carried greater risk in these investigations.^7^, ^12^, ^13^, ^20^, ^46^, ^51^ However, in a study of young Californians affected by wildfires,^41^ being female was significantly associated with PTG and neither age or sex were related to PTS symptoms in a study of young people impacted by flooding in Nashville, Tennessee.^52^

PTG, the capacity to experience personal growth in the wake of trauma, has also been examined (in addition to symptoms of anxiety) as it relates to deliberate and intrusive rumination in childhood survivors of EWEs.^53^, ^54^ Briefly, intrusive rumination is distinct from deliberate rumination in that the thought process is not intentional or controlled.^54^ Rather, negative thoughts related to the traumatic event invade without regulation. Deliberate rumination describes a process in which the affected individual re‐examines the traumatic events with the intention of learning from the experience and reframing it as a catalyst for personal growth. While results are somewhat mixed,^54^ intrusive rumination was found to hamper PTG in at least a handful of studies.^55^, ^56^ Similarly, Abel et al. found that excessive and persistent rehashing of negative experiences with a caregiver (i.e., ‘coreminiscing’) was associated with heightened child anxiety in young people after the second most intense tornado on the Enhanced Fujita Scale.^53^ Conversely, deliberate rumination was associated with PTG in a study of middle schoolers who survived a tornado in Yancheng, China. This result is consistent with the broader literature on the utility of deliberate rumination in trauma survivors.^54^

Disaster‐related loss is also a recurring theme in the literature, with greater loss being related to negative outcomes.^20^, ^52^ Potential consequences of an EWE include property damage or loss of one’s home, loss of services/resources, disruption of social support networks due to displacement, interruption of schooling, injury or death of family/friends, and myriad other personal losses. Disaster exposure – a term that appears relatively frequently in the literature, often as a risk factor for negative mental health outcomes – overlaps conceptually with disaster‐related loss.^12^, ^41^, ^46^, ^52^, ^57^ For example, the Exposure to Tornado Scale^46^ includes items related to physical injuries, property damage, and disruption of basic services (electricity, water). Paul et al. used this scale to determine the relationship between tornado exposure and PTSD symptoms in 2000 adolescents and found a positive, significant relationship (*p*<0.001).^46^ Specifically, as tornado exposure increased so did PTSD symptoms. An investigation by Sheerin et al. yielded a similar result, as tornado severity (amongst exposed adolescents) was associated with elevated baseline symptoms of PTS.^57^ Several studies focused on childhood hurricane and flood survivors corroborate these findings and provide further evidence of a strong relationship between the degree of exposure to the EWE and undesirable consequences.^12^, ^41^, ^52^

Finally, as previously discussed, the presence of a parent/caregiver during an EWE appears to confer protection. It is, therefore, intuitive that the absence of a parent may place a child at an elevated risk of a complicated or incomplete mental health recovery process.^20^, ^24^ Other factors that have been implicated as potential risk factors for negative mental health outcomes include pre‐disaster levels of negative life events and negative friendship interactions,^52^ having experienced interpersonal violence,^30^ lower household income,^46^ parental injury,^20^ and exposure to community violence.^58^

## IMPLICATIONS FOR SCREENING AND TREATMENT

While up to 71% of children report posttraumatic symptoms in the aftermath of disasters,^10^, ^59^ only 5% to 33% access counseling.^60^ While barriers to and facilitators of post‐disaster treatment are not the focus of this review, we would like to highlight some prominent themes in the literature.

Several investigators intimated that participant characteristics – sociodemographic, cultural, and exposure‐related – should be factored into assessment and treatment approaches as they affect a child’s risk level and chances of recovery.^46^, ^61^, ^62^ Others communicated the need for interventions at the individual, family, and community levels,^41^, ^46^ while Lai et al. advocated policies that include comprehensive mental health screenings for child EWE survivors.^63^ These same authors also observe that in the wake of disasters, communities and families are often consumed with the provision of basic needs – such as reopening schools and ensuring physical safety – rather than on mental health issues.^63^ They also noted that as time elapses and a return to ‘normality’ seems within reach, parents may become more capable of tending to their children’s emotional needs.^63^ The consuming nature of securing basic resources in the immediate post‐disaster period should be a consideration in screening and treatment planning and lends credence to the call for family‐focused (rather than individual‐level) interventions.^63^

Moreover, despite documented implementation challenges, there are published reports indicating the value in post‐disaster screening and treatment for both parents and children.^60^, ^64^, ^65^, ^66^ In fact, in a study by Poulsen et al., parents of children who participated in post‐disaster screening and treatment reported being largely satisfied with the process.^66^ This is significant as parental satisfaction has been associated with adherence to child treatment plans.^64^ Further, Cohen et al. explain that a robust screening process aimed at identifying the most vulnerable individuals is a necessary predecessor to all clinical approaches.^64^

Related to innovative treatments, the therapeutic benefit of promising coping techniques, such as dispositional mindfulness, deliberate rumination, and positive reappraisal, merit further investigation.

## SUMMARY

EWEs are increasing in intensity and frequency as part of the climate crisis.^6^ While changes in weather patterns will have an impact on all of humanity, children and individuals of lower socioeconomic status will be most affected. It is also important to note that children are particularly vulnerable due to a lack of agency. Specifically, they cannot control their exposures because parents, teachers, or other caregivers make many choices on their behalf, and these choices shape their environment.^67^ PTS is the most common and frequently studied mental health consequence in child survivors of EWEs^9^ and it is often comorbid with depression and/or anxiety.^12^, ^68^, ^69^

While social support, parental presence during the disaster, and exposure level are relatively well studied and appear to be strongly linked to child mental health outcomes, the role of most other variables is somewhat inconclusive because of mixed or limited evidence. Regarding the persistence of psychological symptoms, there is ample evidence – across EWE type – that long‐term mental health effects are a possibility in the aftermath of an EWE.^33^, ^34^, ^35^ However, recovery is highly individualized and influenced by numerous factors including resiliency,^7^ level and type of social support,^47^ degree of personal loss,^52^ and chronicity of the event.^32^ It is also important to note that not all EWEs are comparable in terms of their potential impact on the individual, society, and the region as a whole. Puerto Rico, for example, has experienced compounding disasters including successive hurricanes in 2017, ongoing seismic activity, and, presently, COVID‐19, making it difficult for its citizens to recover fully.^70^ In a review of the related literature, Cianconi et al. make a key distinction between the psychological consequences of acute, isolated weather events (i.e. a single tornado) versus a prolonged event or series of events impacting one geographic location.^71^

In this review, we have attempted to synthesize the recent literature as it pertains to EWEs and children’s mental health. Therefore, the framing and content of the discussion is heavily influenced by the literature from 2010 and beyond; this approach has corresponding advantages and disadvantages. On a more obvious note, the older literature is underrepresented. However, the timeframe for the review represents a time of heightened climate consciousness and scientific interest. In fact, the Yale Program on Climate Change Communication asserts that, ‘Americans increasingly understand that climate change harms human health.’^72^ This understanding of the broader public health implications of climate change may prove beneficial in terms of the synthesis, interpretation, and application of recent data. Finally, there is a strong focus on the adolescent population in the related literature. While this also has its advantages, our understanding of EWE effects in younger children may be incomplete.

## Data Availability

Data available on request due to privacy/ethical restrictions.
